# Comprehensive Review of the Cardinal Ligament

**DOI:** 10.7759/cureus.2846

**Published:** 2018-06-20

**Authors:** Seif Eid, Joe Iwanaga, Rod J Oskouian, Marios Loukas, R. Shane Tubbs

**Affiliations:** 1 Anatomical Sciences, St. George's University, St. George's, GRD; 2 Seattle Science Foundation, Seattle, USA; 3 Neurosurgery, Swedish Neuroscience Institute, Seattle, USA; 4 Neurosurgery, Seattle Science Foundation, Seattle, USA

**Keywords:** cardinal ligament, transverse cervical ligament, pelvic prolapse, uterus, parametrium

## Abstract

The cardinal ligament is thought to be one of the important structures in providing support and stabilization for the pelvic organs e.g., the uterus. However, many discrepancies exist in the literature regarding terminology, anatomy, and histology. The cardinal ligament attaches the lateral side of the vagina and cervix to the lateral pelvic wall, which provides support to the vagina and cervix. Studies have shown variable findings in the collagen content and distribution in the cardinal ligaments of women with a prolapsed uterus. Uterine vessels and the branches of the inferior hypogastric plexus travel in the cardinal ligament, which is of great importance during pelvic surgeries. Cervical cancer may lead to metastatic spread to the lymph nodes juxtaposed to the cardinal ligaments. This review aims to highlight the clinical significance and surgical importance of the cardinal ligament with a comparison with previous studies.

## Introduction and background

**Figure 1:**
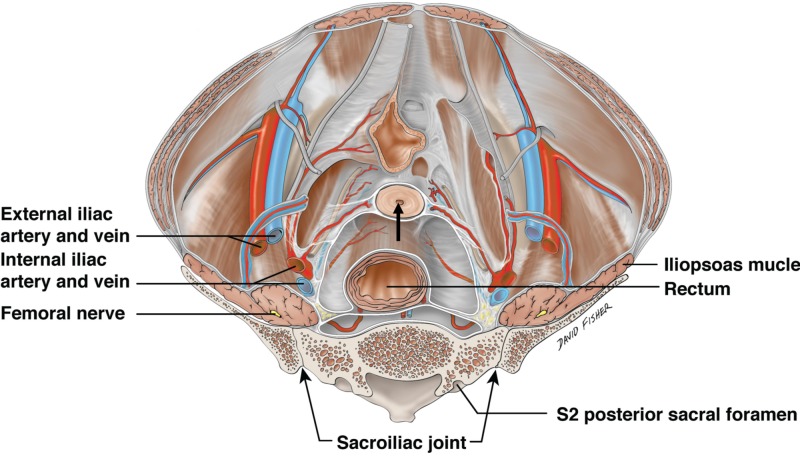
Superior view of the female pelvis external to the peritoneum Note the lower uterus cut in cross-section (arrow) and being suspended laterally by the cardinal ligaments carrying the uterine arteries. Also note the ureters just inferior to the uterine arteries and their relationship to the cardinal ligaments.

The support of the pelvic organs is contributed to by the cardinal, uterosacral, and uterovesicular ligaments and the pelvic floor musculature. Of these, known by several names, the cardinal ligament (Figure 1) is a structure that provides the support and stabilization of the cervix and the upper vagina to the pelvic wall. It is important to note that the cardinal ligament is not a skeletal ligament and contains arteries, veins, and nerves. Various inconsistencies appear in the literature regarding this structure due to the variability and complexity of pelvic pathology. This review aims to highlight the anatomical and clinical significance of the cardinal ligament to provide a better understanding for clinicians.

## Review

History

In 1880, Josef Kocks was the first to name this structure the cardinal ligament [1]. Later, in 1895, Mackenrodt named it the transverse cervical ligament and since then, it often takes on the eponym, Mackenrodt’s ligament [2]. The literature contains different terminologies and descriptions of the cardinal ligament [3], although the most recent Terminologia Anatomica term for this structure is the cardinal ligament. Interestingly, some have even questioned the existence of the cardinal ligament as a separate entity. For example, Moritz reported that this ligament is a continuation of the parametrium, which can be separated only artificially by dissection [4].

Anatomy

Although several differences exist regarding the identification of the cardinal ligament, the functional ability of this ligament to provide pelvic support is agreed upon. The cardinal ligaments are not skeletal ligaments composed of regular dense connective tissue, rather these are mesenteric structures consisting of loose connective tissue and smooth muscle [5]. Range and Woodburne [6] described the cardinal ligament as a mesenteric condensation composed of loose areolar connective tissue surrounded by blood vessels, nerves, and lymphatics, which can only be visually distinguished when the uterus is retracted to the contralateral side. The cardinal ligaments originate from the lateral pelvic wall and attach on the lateral cervix and vagina. These ligaments collaborate with the uterosacral ligaments and the pelvic musculature to provide support to the pelvic organs and to prevent prolapse. The cardinal ligament is vertically oriented while the uterosacral ligaments are dorsally oriented in the standing position, which collectively provides apical support for the uterus and vagina [7]. As the uterine vessels and inferiorly located ureter are related to the cardinal ligament, they can be injured during pelvic surgery where the cardinal ligament is manipulated. The ureter is approximately 2 cm lateral to the supravaginal region of the cervix [5]. Fibers from the uterovaginal plexus derived from the inferior hypogastric plexus travel in the cardinal ligament, which provides innervation to the clitoris, vestibular glands, and vaginal wall [8]. A cadaveric study revealed that the cardinal ligament was continuous with the vesicohypogastric fascia and that the uterine artery can be found in the superior region of the cardinal ligament while the uterine vein or middle vesical artery/vein can be found in its inferior region [9]. Magnetic resonance imaging (MRI) has aided in constructing 3D models that demarcated the cardinal and uterosacral ligaments and that demonstrated that the cardinal ligament is longer and more curved than the latter [7].

Histology

The cardinal ligament can be subdivided based on histological characteristics. Range and Woodburne [6] revealed variable arrangement in collagen fibers and inconsistent cellular and vascular components in different regions of the cardinal ligament on microscopic examination. Another histologic study revealed increased expression of collagen III and tenascin with decreased elastin in the cardinal ligaments of women with a prolapsed uterus [10]. On the other hand, some studies have reported decreased levels of collagen in women with pelvic organ prolapse and urinary incontinence [11-13]. Salman et al. [14] found that the collagen fibers were more densely arranged and were thicker in women without uterine prolapse.

Kato et al. [15] revealed the presence of branches of the pelvic splanchnic nerves in the dorsal aspect of the cardinal ligament, which was separated by the vascular portion by loose connective tissue. Some researchers were able to demonstrate nerve content by immunofluorescence techniques and found that the cardinal ligament consisted of many free nerve fibers with a significant concentration of these fibers in the lateral third of the ligament [16]. Another study of patients with cervical cancer discovered an association between high-risk disease and the presence of metastatic disease in the lymphatics found in the vascular region of the cardinal ligament along with pelvic lymph nodes [17]. This would suggest that the complete removal of the vascular portion of the cardinal ligament might be indicated.

Clinical associations

Although many disagreements exist regarding the terminology, the clinical implications of the cardinal ligament related to disorders of the pelvic floor and cervical malignancy must be acknowledged. It is important to note that the pelvic floor musculature and the pelvic fascia mutually contribute to the stability of the pelvic floor [18]. The levator ani muscle provides support to the pelvic organs and consists of the puborectalis, pubococcygeus, and iliococcygeus muscles [19]. In a study using 3D models from MRI, authors have developed four patterns of levator ani avulsion pathology that was associated with pelvic organ prolapse. This study also revealed that there was a significant difference in the morphology of the puborectalis and pubococcygeus parameters in women with prolapse compared to the control group while there was no difference in the morphology of the iliococcygeus muscle [19]. Otcenasek et al. [18] performed a similar study to classify the causes of pelvic prolapse and revealed that prolapse may occur due to a defect of the fascia and can be either partial or complete tears while the muscle defects can be variable. Some authors have identified the cardinal ligament as a structure consisting of the lateral parametrium observed from the dissected paravesical space while the transverse cervical ligament is observed from the dissected pararectal space [9]. Yabuki describes the cardinal ligament and the transverse cervical ligament as two different structures that comprise the supporting system of the pelvis [20]. He emphasizes that the cardinal ligament represents only the medial side of the supporting system while the transverse cervical ligament is the lateral continuation of the cardinal ligament that forms a complex with the lateral ligament of the rectum and the umbilical ligament [20].

From a surgical perspective, the cardinal ligament still remains a vital landmark, as it is one of the pedicles to be secured during a hysterectomy. According to Cruikshank and Kovac [21], the traction and cutting of the cardinal ligament are the main factors that affect ureter movement during vaginal hysterectomy. This leads to the movement of the ureter out of the operative field, thereby protecting the ureter from possible injury. One recent study evaluating different guideline protocols proposed that the possibility of pelvic prolapse after hysterectomy may decrease by the suspension of the vaginal vault to the cardinal and uterosacral ligaments [22]. Another important surgical consideration is the prediction of the uterine anatomy based on the cervical position, which is an important consideration for several gynecological procedures. Fidan et al. [23] revealed that the anatomy of the uterus and adjacent tissues can be predicted by pelvic examination and confirmed with ultrasonography. This study revealed that the uterus was anteverted in 90% of cases with the cervix being in the posterior fornix position while uterine anteversion was present in 64.2% of cases with an anterior cervix [23].

In addition, the cardinal ligament is also involved in radical hysterectomy for cervical cancer due to the lymphatic drainage of the cervix. Girardi et al. [24] revealed that both positive and negative pelvic nodes can be found in variable locations along the parametrium, even at the most lateral region near the pelvic side wall. This study also revealed that positive parametrial lymph nodes are strongly associated with the presence of positive pelvic lymph nodes and a higher recurrence rate of cervical cancer [24]. This may suggest more extensive resection of the cardinal ligament to ensure a disease-free state. However, one must also consider possible damage to the pelvic plexuses traveling in these ligaments to avoid urinary bladder, rectal, and sexual complications that may arise when these ligaments are resected during a radical hysterectomy [25]. It was previously reported that the nerve content is variable along the length of the cardinal ligament; however, there is an increased nerve content in its lateral thirds from the pelvic wall [16].

Ramanah et al. [26] analyzed the literature and describes the cardinal and uterosacral ligaments as “visceral ligaments,” which contain blood vessels, nerves, connective tissue, and adipose tissue. These authors described the origin of the internal iliac artery as the proximal insertion point and the cervix and upper vagina as the distal insertion point of the cardinal ligament [26]. Another review also revealed similar findings and inconsistencies relating to the description and characteristics of the cardinal ligament [27]. This signifies the importance of obtaining a consistent description of these ligaments in order to better understand the pathologies of the pelvic floor.

## Conclusions

The aim of this review was to highlight some of the disagreements and to provide a better understanding of the nature of the cardinal ligament. Due to the complexity of the pelvic floor, further research is warranted to clear some of the inconsistencies in the literature. Despite the differences in terminology, the clinical and surgical significance of this ligament is paramount in providing ideal patient care.
